# Ciproxifan, a histamine H_3_ receptor antagonist, reversibly inhibits monoamine oxidase A and B

**DOI:** 10.1038/srep40541

**Published:** 2017-01-13

**Authors:** S. Hagenow, A. Stasiak, R. R. Ramsay, H. Stark

**Affiliations:** 1 Heinrich Heine University Duesseldorf, Institute of Pharmaceutical and Medicinal Chemistry, Universitaetsstr. 1, 40225 Duesseldorf, Germany; 2 Department of Hormone Biochemistry, Medical University of Lodz, Zeligowskiego 7/9, Pl 90-752 Lodz, Poland.; 3 Biomedical Sciences Research Complex, University of St Andrews, North Haugh, St Andrews KY16 9ST, United Kingdom

## Abstract

Ciproxifan is a well-investigated histamine H_3_ receptor (H3R) inverse agonist/antagonist, showing an exclusively high species-specific affinity at rodent compared to human H3R. It is well studied as reference compound for H3R in rodent models for neurological diseases connected with neurotransmitter dysregulation, e.g. attention deficit hyperactivity disorder or Alzheimer’s disease. In a screening for potential monoamine oxidase A and B inhibition ciproxifan showed efficacy on both enzyme isoforms. Further characterization of ciproxifan revealed IC_50_ values in a micromolar concentration range for human and rat monoamine oxidases with slight preference for monoamine oxidase B in both species. The inhibition by ciproxifan was reversible for both human isoforms. Regarding inhibitory potency of ciproxifan on rat brain MAO, these findings should be considered, when using high doses in rat models for neurological diseases. As the H3R and monoamine oxidases are all capable of affecting neurotransmitter modulation in brain, we consider dual targeting ligands as interesting approach for treatment of neurological disorders. Since ciproxifan shows only moderate activity at human targets, further investigations in animals are not of primary interest. On the other hand, it may serve as starting point for the development of dual targeting ligands.

Ciproxifan (cyclopropyl 4-(3-(1*H*-imidazol-4-yl)propyloxy)phenyl methanone) is a well characterized species-specific histamine H_3_ receptor (H3R) inverse agonist/antagonist (Fig. 1). It shows exclusively high affinity at rodent H3R in a sub-nanomolar range (K_i_ (rH3R) = 0.4–6.2 nM and K_i_ (mH3R) = 0.5–0.8 nM), while binding to human H3R is only moderate (K_i_ = 46–180 nM) with negligible selectivity e.g. over human adrenergic α_2A_ and α_2C_ receptors^1^,^2^,^3^ (Table 1). Ciproxifan’s inverse agonism/antagonism at histamine H_3_ receptors is manifested in improvement of wakefulness and attention *in vivo*^4^,^5^. It is commonly used as reference H3R antagonist, e.g. in rodent models studying cognitive impairment^6^, Alzheimer’s disease^7^ or attention deficit hyperactivity disorder (ADHD)^8^. It was also tested in animal models for schizophrenia^9^, sleeping disorders^10^ or most recently autism^11^.

The H3R (for review, e.g. see Sander *et al*.^5^ or Gemkow *et al*.^12^), a G-protein coupled receptor displaying constitutive activity (basal activity without binding of an agonist), inhibits the release of several neurotransmitters like dopamine, histamine, serotonin or acetylcholine^4^. In consequence, inverse agonism/antagonism of the H_3_ receptor leads to accelerated release of mentioned neurotransmitters which is why H_3_ receptor inverse agonists/antagonists like ciproxifan are recognized as promising therapeutics for treatment of several neuropathological diseases^13^. During a screening for monoamine oxidase A (MAO A) and B (MAO B) inhibitors, ciproxifan was found to be an inhibitor for both enzyme isoforms. MAOs are expressed in neurons and glial cells, localized in the cell on the outer membrane of mitochondria and critically involved in degradation of neurotransmitters in the brain. In humans MAO A is predominantly found in adrenergic, catecholaminergic and dopaminergic neurons and deactivates serotonin, dopamine, norepinephrine and epinephrine. Human MAO B participates in dopamine degradation and is mainly expressed in serotonergic neurons and glial cells^14^,^15^. Therefore, MAO inhibitors are frequently investigated for treatment of depression and Parkinson’s disease^16^. In this study, we further investigated ciproxifan’s capability to inhibit human MAO A and MAO B *in vitro* by determination of IC_50_ values and reversibility of its inhibition.

## Results

### IC_50_ determinations for human MAO

**Figure 1:**
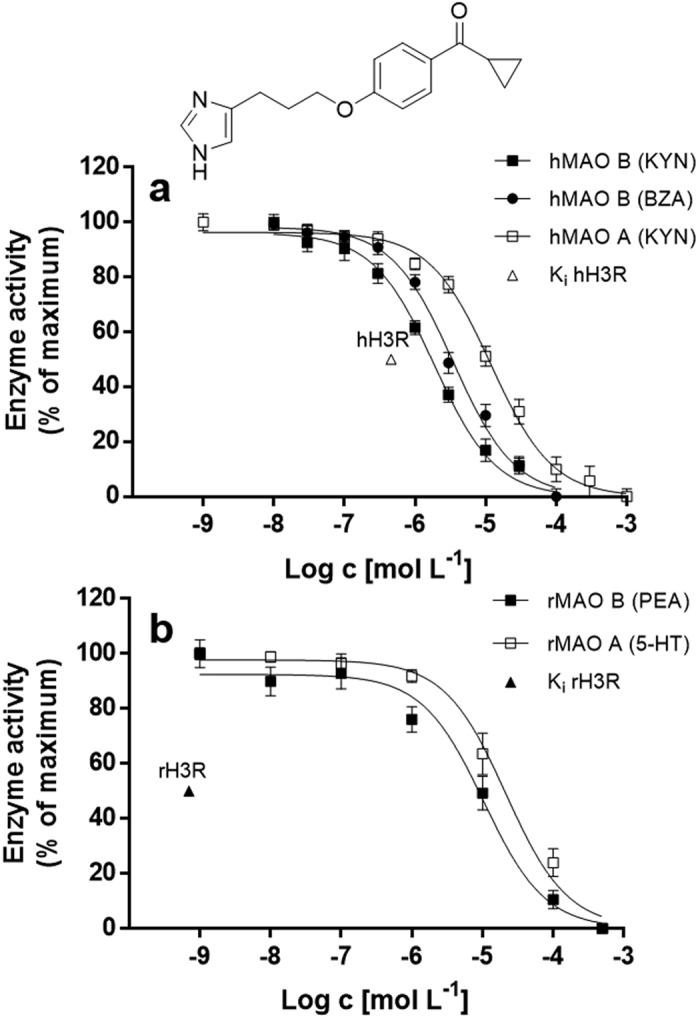
(**a**) Inhibition curves for ciproxifan obtained with a spectrophotometric assay using human recombinant membrane-bound MAO A and MAO B. (**b**) Inhibition curves for ciproxifan in rat brain MAO A and MAO B measured radiometrically. Kynuramine (KYN, **a**) or serotonin (5-HT, **b**) were used as MAO A substrates. Kynuramine (KYN, **a**), benzylamine (BZA, **a**) or phenylethylamine (PEA, **b**) were used as MAO B substrates. Data represent mean ± s.e.m. of at least n = 3 independent experiments each performed at least in duplicates (global fit). The K_i_ values of ciproxifan for human histamine H_3_ receptors (hH3R, ∆) and rat histamine H_3_ receptors (rH3R, ▲) are indicated in the graphs^1^.

The IC_50_ values of ciproxifan for human membrane-bound MAO (hMAO) were measured spectrophotometrically using kynuramine (KYN) and benzylamine (BZA) as MAO B substrates, while for MAO A only KYN was used. We found IC_50_ values for ciproxifan in a micromolar range (IC_50, MAO A_ = 11 μM and IC_50, MAO B_ = 2 μM), showing an about 5-fold higher preference for MAO B (IC_50, MAO B_/IC_50, MAO A_ = 0.2) (Fig. 1, Table 2).

*l*-Deprenyl, clorgyline, safinamide and moclobemide were tested as reference compounds using the same spectrophotometric method. The irreversible MAO B selective inhibitor *l*-deprenyl showed an IC_50_ value of 37 nM for MAO B (Table 2). For clorgyline, an irreversible MAO A selective inhibitor, an IC_50_ value of 8 nM for MAO A were found. The reversible inhibitors safinamide and moclobemide gave IC_50_ values of 49 nM (MAO B) and 568 μM (MAO A), respectively (Table 2).

### IC_50_ determinations for rat brain MAO

The IC_50_ values of ciproxifan for rat brain MAO (rMAO) were obtained radiometrically using serotonin (5-HT) and phenylethylamine (PEA) as MAO A and MAO B substrates, respectively. Similar to hMAO, ciproxifan displayed IC_50_ values in the micromolar concentration range (IC_50, MAO A_ = 38 μM and IC_50, MAO B_ = 15 μM), again with slight preference for MAO B (IC_50, MAO B_/IC_50, MAO A_ = 0.4) (Table 2).

### Reversibility of human MAO inhibition

In order to determine whether ciproxifan shows a reversible or irreversible inhibition type, dilution experiments using the spectrophotometric assay were performed, where hMAOs were preincubated with ciproxifan (10 × IC_50_). After preincubation probes were diluted 100-fold, measured at saturated substrate conditions and the remaining enzyme activity was compared to that of MAO preincubated without ciproxifan. For both hMAO isoforms no considerable decrease in enzyme activity after preincubation with ciproxifan compared to control (set to 100%) were observed, suggesting a reversible inhibition type (Table 2). The remained enzyme activities for hMAO A and hMAO B preincubated with ciproxifan were 107.7 ± 3.4% and 91.4 ± 9.7%, respectively. In order to verify the test procedure, *l*-deprenyl was tested in the same manner showing decreased remaining enzyme activity of hMAO B (51.1 ± 2.9%) after preincubation.

## Discussion

Ciproxifan is frequently used as the reference histamine H_3_ receptor (H3R) antagonist in rodent models for neurological diseases like cognition^6^, Alzheimer’s disease^7^ or sleep-wake disorders^10^,^17^, because of its explicit high affinity and efficacy in rodent H3R (K_i_ < 1.0 nM) which is about 30- to 100-fold lower than that at the human H3R. In our study we showed an additional property of ciproxifan. It inhibits human and rat MAO A and MAO B reversibly in a micromolar concentration range with a slight preference for MAO B.

We consider a combined activity pattern of ligands at H3R and MAO as new interesting approach for the treatment of neurological diseases. These are often associated with a neurotransmitter dysregulation, assumed to be adjustable by H3R blockade^12^. Neurotransmitter levels could be also modulated by inhibition of their degradation. MAOs are enzymes involved in oxidative deamination of neurotransmitters in neuronal cells after reuptake from the synaptic cleft. Therefore, deactivation of MAO A or MAO B is a principle well established in therapy of neurological disorders like depression and Parkinson’s disease^18^. Additionally, MAOs are thought to promote oxidative stress when highly expressed in neuronal tissues. This can force increased neuronal cell death, a condition observed in Alzheimer’s disease^19^. Thus, we hypothesize that reversible or more probably irreversible MAO inhibitors and H3R inverse agonists/antagonists can have overlapping pharmacological utilities, suggesting ligands, interacting with both targets, as promising candidates for treatment of neurodegenerative diseases. Concerning ciproxifan, which displays IC_50_ values for both hMAO isoforms only in a micromolar range, we found its inhibition far too low for therapeutic efficacy in humans, which is anyway limited by its low affinity at human H3R. For example, safinamide, a MAO B selective reversible inhibitor most recently approved as first add-on treatment of Parkinson’s disease, is active in submicromolar concentration ranges (IC_50_ = 0.048–0.112 μM for hMAO B; IC_50, MAO B_/IC_50, MAO A_ < 0.001^20^,^21^,^22^). Additionally, imidazole-containing drugs like ciproxifan are potential inhibitors of cytochrome P450 enzymes by coordination of the heme iron atom^23^. So, it may only serve as prospective starting point for investigation of dual targeting ligands in the future. Since ciproxifan is frequently used in rodent models for several neurological diseases, its MAO inhibition should be taken into consideration in retrospect or in the future using rodent models as possible accompanying effect. Ciproxifan has about four orders of magnitude higher activity at rH3R compared to the in-cell target rMAO. However, with a calculated log P value of 2.76^24^ we assume good membrane penetration by ciproxifan^23^. Additionally, it could be shown that ciproxifan can reach brain concentrations up to approximately 10 μM when applied i.p. to rats^17^, evidencing that partial rMAO inhibition is at least conceivable under test conditions.

Taken together, the moderate and reversible MAO inhibitory properties of ciproxifan are interesting newly described properties which may interact with some previous animal screening data at high ciproxifan dosages. It can still be taken as a reference H3R antagonist, but its MAO A and MAO B inhibitory properties may be considered in retrospect on high dosage screenings.

Nevertheless, ciproxifan may serve as starting point for the design of dual targeting ligands combining H_3_ receptor inverse agonism/antagonism and MAO inhibition, possibly favourable in treatment various neurological diseases. Since ciproxifan inhibited rat brain MAO also in micromolar concentration ranges, its activity has to be take into consideration in the future as a possible accompanying effect, when using it in rat models for neurological diseases. In retrospect, some of its effects explored in different species were probably contributed by its MAO A/B inhibitory properties.

## Methods

All authors confirmed that all methods were carried out in accordance with relevant guidelines and regulations.

### Spectrophotometric IC_50_ determination using human MAO

Enzyme studies were carried out using human recombinant membrane-bound MAO A and MAO B (Sigma-Aldrich, St. Louis, MO). Pipetting of assays were fully automated using a pipetting robot in a total assay volume of 100 μL or performed manually in a total assay volume of 200 μL. IC_50_ values were obtained by measuring enzymatic conversion rates at inhibitor concentrations between 10^−9^ M and 10^−3^ M in the presence of kynuramine (K_M_ = 40 μM for MAO A and K_M_ = 25 μM for MAO B) or benzylamine (K_M_ = 165 μM) using 2 × K_M_ substrate concentrations, while reactions were started by addition of MAO A (10 ng μL^−1^) or MAO B (12.5 ng μL^−1^). For optimal enzyme activity all assays were carried out under potassium phosphate buffered conditions (50 mM, pH = 7.4). Initial velocities were determined spectrophotometrically by a microplate reader at 30 °C by following product formation of 5-hydroxyquinoline and benzaldehyde at 316 nm and 250 nm, respectively, over a period of at least 30 minutes (interval of 20–30 seconds). Initial velocities, expressed as mAU min^−1^, were obtained from the linear phase of product formation (see Supplementary Information, Fig. 1). Data were analysed using GraphPad PRISM version 6. For IC_50_ determinations initial velocities for each experiment were normalized (expressed as percentage), plotted against inhibitor concentrations and fitted using the non-linear regression “log inhibitor vs. response (three parameters)”. IC_50_ values were determined in at least three independent experiments, each performed at least in duplicates.

### Radiometric IC_50_ determination using rat brain MAO

Wistar male rats were sacrificed, the brains were quickly excised from the skulls, cleaned of residual meninges and frozen on dry ice. For each experiment the crude homogenates prepared from pooled brain from three rats were used. Enzyme activity of MAO A or MAO B was measured using radioactive substrate (PerkinElmer/NEN): serotonin (5-[2-^14^C]-hydroxytryptamine binoxalate) or β-[ethyl-1-^14^C]-phenyl-ethyl-amine hydrochloride (PEA), respectively, with the procedure described by Fowler and Tipton 1981^25^ with some modification according to Gómez *et al*.^26^. IC_50_ values were determined using six different concentrations of ciproxifan between 10^−9^ M and 5 × 10^−4^ M at fixed concentration of substrate (200 μM serotonin or 20 μM PEA). For IC_50_ determinations values were normalized (expressed as percentage), plotted against inhibitor concentrations and fitted using the non-linear regression “log inhibitor vs. response (three parameters)”. IC_50_ values were determined in three independent experiments, each performed in duplicates.

### Reversibility of human MAO inhibition

Reversibility of hMAO inhibition by ciproxifan was assessed by dilution experiments. Supplied MAOs (5 mg mL^−1^ in potassium phosphate 100 mM, sucrose 0.25 M, EDTA 0.1 mM, glycerol 5%) were preincubated with inhibitor (10 × IC_50_) or water at 30 °C for 15 minutes in a water bath. The inhibitor volume represented one-tenth of the total preincubation volume. After preincubation, probes were 100-fold diluted with potassium phosphate buffer (100 mM, pH = 7.4) to yield a final concentration of 12.5 ng μL^−1^ for the enzyme. Enzymatic conversion rates were determined in the presence of kynuramine for hMAO A and benzylamine for hMAO B at saturating substrate concentrations (10 × K_M_) under potassium phosphate buffer conditions (50 mM, pH = 7.4). Spectrophotometric measurements were carried out as described for IC_50_ determinations over a period of at least 30 minutes for hMAO A and hMAO B, respectively (interval of 20–30 seconds). Initial velocities, obtain from the linear phase of product formation, of hMAO preincubated with inhibitor (10 × IC_50_) were compared to hMAO preincubated with water (control) to define reversible or irreversible inhibition mode (see Supplementary Information, Fig. 2). Initial velocities, expressed as mAU min^−1^, were normalized and given as percent of control.

## Additional Information

**How to cite this article**: Hagenow, S. *et al*. Ciproxifan, a histamine H_3_ receptor antagonist, reversibly inhibits monoamine oxidase A and B. *Sci. Rep.* **7**, 40541; doi: 10.1038/srep40541 (2017).

**Publisher's note:** Springer Nature remains neutral with regard to jurisdictional claims in published maps and institutional affiliations.

## Supplementary Material

Supplementary Informations

## Figures and Tables

**Table 1:** Published affinity data for ciproxifan.

Receptor	Ki [nM]	Receptor	K_i_ [nM]
rH_3_R	0.4–6.2^2^,^3^,^27^,^28^,^29^	gpβ_1_^b^	12589^2^
mH3R	0.5–0.8^30^	gp M_3_^c^	3162^2^
mkH_3_R	41^27^	gp5-HT_3_^c^	>3,162^2^
hH_3_R	46–180^1^,^3^,^27^	gp5-HT_1B_^d^	>10,000^2^
hH_1_R	>10,000^3^	r5-HT_1_	16598^31^
hH_2_R	>10,000^3^	r5-HT_2A_^e^	15848^2^
hH_4_R	1862^3^	r5-HT_3_	302^3^
hα_2C_	63^3^	r5-HT_4_^f^	>1,995^2^
hα_2A_	43^3^		
rα_1D_^a^	3,981^2^		

gp = guinea pig, h = human, m = mouse, mk = monkey, r = rat.

^a^rat aorta.

^b^guinea pig atrium.

^c^guinea pig ileum.

^d^guinea pig iliac.

^e^rat tail.

^f^rat esophagus.

**Table 2:** IC_50_ values and type of inhibition for ciproxifan, l-deprenyl, clorgyline, safinamide and moclobemide using kynuramine (KYN) or serotonin (5-HT) and benzylamine (BZA) or phenylethylamine (PEA) as MAO A and MAO B substrates, respectively.

	IC_50_ [μM] ± s.e.m (n)			IC_50_ [μM] ± s.e.m (n)		Inhibition Type
	hMAO			rMAO		
	A	B		A	B	
Substrate	KYN	BZA	KYN	5-HT	PEA	
Ciproxifan	11.4 ± 1.2 (5)	4.3 ± 0.7 (5)	2.1 ± 0.3 (9)	37.5 ± 0.2 (3)	15.4 ± 0.3 (3)	Reversible
Safinamide	n.d.	n.d.	0.049 ± 0.001 (4)	n.d.	n.d.	Reversible^20^
Moclobemide	568 ± 115 (3)	n.d.	n.d.	n.d.	n.d.	Reversible^32^
*l*-Deprenyl	29.6 ± 3.9 (9)	n.d.	0.037 ± 0.004 (5)	n.d.	n.d.	Mixed/Irreversible^33^
Clorgyline	0.008 ± 0.001 (4)	n.d.	1.3 ± 0.2 (4)	n.d.	n.d.	Mixed/Irreversible^33^

IC_50_ values are given as means ± standard errors of means (s.e.m.) of n independent experiments, each performed at least in duplicates.

n.d. = not determined, *l*-deprenyl IC_50_ = 0.036 μM^33^, clorgyline IC_50_ = 0.0065 μM^33^, safinamide IC_50_ = 0.048 μM^20^, moclobemide IC_50_ = 361 μM^32^.
