# Supplementary material for: Ciproxifan, a histamine H3 receptor antagonist, reversibly inhibits monoamine oxidase A and B
Source: Sci Rep. 2017 Jan 13;7:40541. doi: 10.1038/srep40541 (PMC5233962; doi:10.1038/srep40541)
Supplement: Supplementary Informations [file srep40541-s1.pdf]

Supplementary Informations

Ciproxifan, a histamine H_3 receptor antagonist, reversibly inhibits monoamine oxidase A and B

S. Hagenow[1], A. Stasiak[2], R. R. Ramsay[3] and H. Stark[1]*

[1] Heinrich Heine University Duesseldorf, Institute of Pharmaceutical and Medicinal Chemistry, Universitaetsstr. 1, 40225 Duesseldorf, Germany; e-mail: stark@hhu.de, Fax +49 211 8113359, phone: +49 211 8110478.

[2] Department of Hormone Biochemistry, Medical University of Lodz, Poland Zeligowskiego 7/9, Pl 90-752 Lodz.

[3] Biomedical Sciences Research Complex, University of St Andrews, North Haugh, St Andrews KY16 9ST, United Kingdom.

*Correspondence to stark@hhu.de

Spectrophotometric IC_{50} determination using human MAO

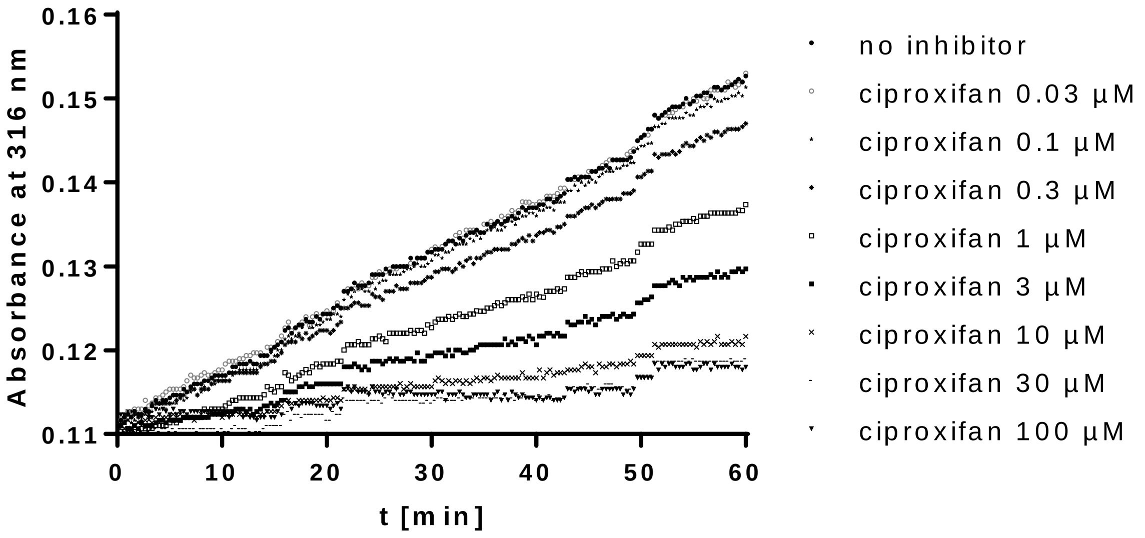

Figure 1. Kinetic measurements of one representative experiment of ciproxifan (eight concentrations, 3 x 10^{-11} to 10^{-4} M) with MAO B using kynuramine (2 x K_M, 50 µM). Enzyme conversion rates were given as mAU min^{-1} over a period of 60 minutes (R^2 >0.97).

Reversibility of human MAO inhibition

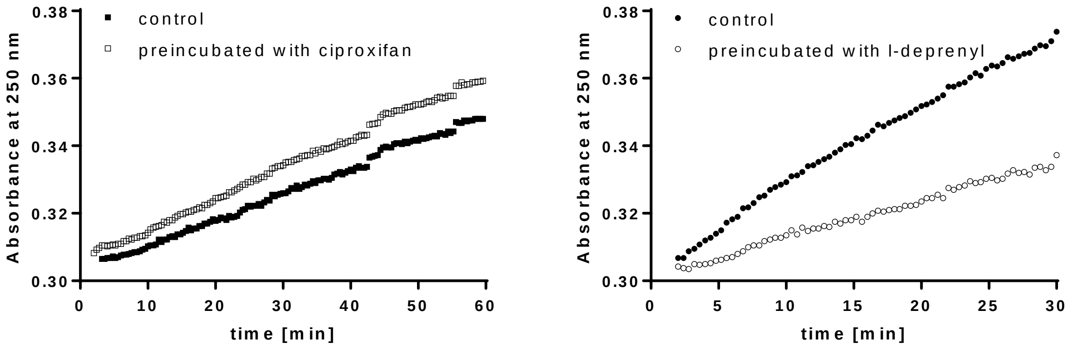

Figure 2. Kinetic measurements of reversibility studies for ciproxifan (left) and L-deprenyl (right) with MAO B. Graphs show one representative experiment for ciproxifan and l-deprenyl each. MAO B were preincubated with water (control) and inhibitor (10 x IC_{50}) for 15 minutes at 30°C. After preincubation samples were diluted (100 x) and enzyme conversion rates were measured as mAU min^{-1} (at 215 nm) in the presence of benzylamine (10 x K_M).
